# An amebic protein disulfide isomerase (PDI) complements the yeast PDI1 mutation but is unable to support cell viability under ER or thermal stress

**DOI:** 10.1002/2211-5463.12350

**Published:** 2017-11-29

**Authors:** Rosa E. Mares, Marco A. Ramos

**Affiliations:** ^1^ Facultad de Ciencias Químicas e Ingeniería Universidad Autónoma de Baja California Tijuana Baja California México

**Keywords:** *Entamoeba histolytica*, endoplasmic reticulum stress, functional complementation, oxidative folding, protein disulfide isomerase, *Saccharomyces cerevisiae*

## Abstract

In eukaryotic cells, protein disulfide isomerases (PDI) are oxidoreductases that catalyze the proper disulfide bond formation during protein folding. The pathobiology of the protozoan parasite *Entamoeba histolytica*, the causative agent of human amebiasis, depends on secretion of several virulence factors, such as pore‐forming peptides and cysteine proteinases. Although the native conformation of these factors is stabilized by disulfide bonds, there is little information regarding the molecular machinery involved in the oxidative folding of amebic proteins. Whereas testing gene function in their physiological background would be the most suitable approach, we have taken advantage of the cellular benefits offered by the yeast *Saccharomyces cerevisiae* (as a model of eukaryotic cell) to examine the functional role of an amebic PDI (*Eh*PDI). As the yeast PDI homolog is essential for cell viability, a functional complementation assay was carried out to test the ability of *Eh*PDI to circumvent the lethal phenotype of a yeast PDI1 mutant. Also, its proficiency under stressful conditions was explored by examining the survival outcome following endoplasmic reticulum (ER) stress induced by a reductant agent (DTT) or thermal stress promoted by a nonpermissive temperature (37 °C). Our results indicate that *Eh*PDI is functionally active when physiological conditions are stable. Nonetheless, when conditions are stressful (e.g., by the accumulation of misfolded proteins in the ER compartment), its functionality is exceeded, suggesting an inability to prevent unfolding, suppress aggregation, or assist refolding of proteins. Despite the latter, our findings constitute the initial step toward determining the participation of *Eh*PDI in cellular mechanisms related to protein homeostasis.

**Abbreviations**
5‐FOA: 5‐fluoroorotic acid
ER: endoplasmic reticulum
G‐418: geneticin
PDI: protein disulfide isomerase
SD: synthetic dextrose
UPR: unfolded protein response
YPD: yeast extract/peptone/dextrose

In eukaryotic cells, protein disulfide isomerases (PDI) are oxidoreductases that catalyze the proper disulfide bond formation during protein folding in the endoplasmic reticulum (ER) 1. As a common structural feature, PDI enzymes share the presence of at least one thioredoxin‐like domain 2. Many of these domains comprise the canonical tetrapeptide CxxC as the active site motif, while some others have a derivative sequence, in which one cysteine was substituted by a selenocysteine, serine, or threonine residue 3. So far studied, almost all eukaryotic cells contain a PDI family with a diverse domain structure and function, for instance, 21 for human and five in yeast cells 4, 5.

The pathobiology of the protozoan parasite *Entamoeba histolytica*, the causative agent of human amebiasis, depends on direct cell contact and secretion of virulence factors 6, 7, 8. Although the native conformation of some of the latter is stabilized by disulfide bonds 9, 10, limited knowledge has been gained on the molecular machinery involved in the oxidative folding of amebic proteins. In this regard, we have used *Eh*PDI as a model to study protein folding assisted by an amebic PDI. Structurally, it shows a domain arrangement like those displayed by orthologous proteins of the P5‐like ERp38 subfamily: two functional thioredoxin‐like domains and a C‐terminal ERp29c‐like domain (Fig. 1). Functionally, it exhibits the typical activities showed by functional oxidoreductases: oxidase, reductase, and isomerase. Besides, it can complement the DsbA (oxidase) and DsbC (isomerase) mutation in bacteria 11, 12, 13, 14, 15.

**Figure 1:**
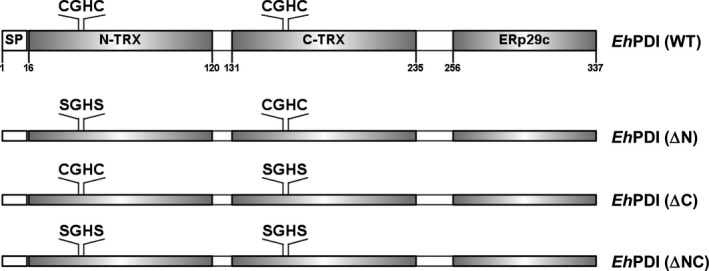
Domain organization of *Eh*PDI (NCBI No. AAU45393). The relative position of each thioredoxin‐like domain, as well as the ERp29c‐like domain, is denoted by numbering in the boundaries of each box. The respective location of the CxxC motif within each thioredoxin‐like domain is also shown. The signal peptide (sp, 15‐residue) is likewise represented. The variants of *Eh*PDI (ΔN, ΔC, and ΔNC) are depicted showing only the corresponding Cys to Ser substitutions.

Although testing gene function in their physiological context is the most desirable strategy, the ability of an orthologous gene to complement the loss‐of‐function mutation in the yeast *Saccharomyces cerevisiae* remains as a reasonable approach 16, 17, 18. Although *S. cerevisiae* has a PDI family comprising five members: PDI1, EUG1, EPS1, MPD1, and MPD2 5, only PDI1 is essential for cell viability 19, 20. Hence, to gain further insights into the functional role of *Eh*PDI, we used a complementation assay to test its ability to circumvent the lethal phenotype associated with the PDI1 mutation in yeast. In addition, its proficiency under stressful conditions was weighted by examining the survival outcome after cell proliferation: (a) in the presence of the xenobiotic DTT (ER), a well‐known ER stress inducer 21, and (b) at nonpermissive temperature (37 °C), a physiological condition recognized as a thermal stress promoter 22.

## Materials and methods

### Reagents

Unless otherwise noted, chemicals and biochemicals were from Sigma‐Aldrich (St. Louis, MO, USA), New England Biolabs (Ipswich, MA, USA), and Qiagen (Germantown, MD, USA).

### Yeast strains and growth conditions

The heterozygous diploid strain BY4743‐YCL043C (MATa/α his3/his3 leu2/leu2 lys2/LYS2 met15/MET15 ura3/ura3 PDI1/pdi1::kanMX) was acquired from Open Biosystems (Huntsville, AL, USA). The haploid strain RSA4‐16, carrying a disruption of the PDI1 locus and maintained viable by the episomal expression of the pdi1 gene, was obtained from the diploid strain by performing a random spore analysis 23. Competent cells were transformed with 1 μg of pCT38, a URA3 plasmid containing the pdi1 transcriptional unit 24. Stable transformants harboring the URA3 plasmid were selected by their ability to grow in the absence of uracil. A single Ura^+^ colony‐forming unit was picked and sporulated in starvation medium. The wall of vegetative cells (tetrads) was digested with β‐glucuronidase, and released spores (haploid cells) were selected in minimal medium lacking uracil. Analysis of auxotrophic markers was used for establishing the genotype. The lethal phenotype of the yeast PDI1 mutant strain, RSA4‐16 [Δpdi1 (pCT38)], was confirmed by the inability to proliferate in the presence of 5‐fluoroorotic acid (5‐FOA; 0.1%), which is converted into the toxic compound 5‐fluorouracil by the URA3 gene product (orotidine‐5′‐monophosphate decarboxylase). The haploid strain W303‐1B Δpdi1/pCT37, carrying a disruption of the PDI1 locus and maintained viable by the episomal expression of PDI1 under the control of the GAL1 promoter (pGAL1‐PDI1), was kindly provided by T. Stevens (University of Oregon, USA) 24. Unless otherwise mentioned, cells were propagated at 30 °C in synthetic medium supplemented with the appropriate amino acids [and 0.2 mg·mL^−1^ geneticin (G‐418), when needed].

### Plasmid constructions

The plasmids used throughout this study are listed in Table 1. Plasmids pRS413 and pRS414 were used as the backbone shuttle vectors for subcloning and protein expression purposes 25. The control plasmid pYScPDI1, carrying a minimized version of the yeast PDI1 transcriptional unit, was constructed by subcloning a 2.2‐kb *Apa*I‐*Sac*II fragment from pCT38 into pRS413. The yeast expression plasmid p13PSXDEL2, containing the promoter and terminator sequences as well as the ER sorting and retention signals, was constructed as pYScPDI1 with the slight modification: The insert was a 0.8‐kb overlapping PCR product in which the corresponding 5′ and 3′ regulatory sequences were assembled and linked by an 18‐bp stretch (comprising the *Bam*HI and *Xho*I cloning sites). *Eh*PDI‐expressing plasmids: pYEhPDI, pYRM05, pYRM06, and pYRM015, having the mature polypeptide sequence (WT, ∆N, ∆C, or ∆NC, respectively; Fig. 1) enclosed by the ER‐targeting signals and controlled by the yeast PDI1 promoter, were obtained by inserting the respective *Bam*HI‐*Xho*I fragment into p13PSXDEL2. Each coding sequence was amplified from the corresponding bacterial expression plasmid 11, 12. Plasmids p14ScPDI1 and p14EhPDI, which, respectively, express YPDI1 or *Eh*PDI (WT), were constructed by inserting the corresponding *Apa*I‐*Sac*II fragment, from pYScPDI1 or pYEhPDI, into pRS414. All molecular cloning techniques were carried out following the standard protocols. The bacterial strain *Escherichia coli* XL1‐Blue MRF′ (Δ(mcrA)183 Δ(mcrCB‐hsdSMR‐mrr)173 endA1 supE44 thi‐1recA1 gyrA96 relA1lac [F′ proAB lacI^q^ZΔM15 Tn10 (Tet^R^)]), obtained from Stratagene, was used routinely as a host for cloning purposes.

**Table 1:** Strains and plasmids used in this study

Plasmids	Relevant features	Reference
pCT38	pRS316 derivative, centromeric, URA3, containing the yeast PDI1 transcriptional unit (from −879 to +160 bp)	23
pRS413	Centromeric, HIS3	24
pYScPDI1	pRS413 derivative, containing the yeast PDI1 transcriptional unit (from −451 to +160 bp)	This study
p13PSXDEL2	pRS413 derivative, containing the yeast PDI1 regulatory sequences (as pYScPDI1) plus the ER‐targeting signals	This study
pYEhPDI	p13PSXDEL2 derivative, expressing *Eh*PDI (WT)	This study
pYRM05	p13PSXDEL2 derivative, expressing *Eh*PDI (∆N)	This study
pYRM06	p13PSXDEL2 derivative, expressing *Eh*PDI (∆C)	This study
pYRM15	p13PSXDEL2 derivative, expressing *Eh*PDI (∆NC)	This study
pRS414	Centromeric, TRP1	24
p14ScPDI1	pRS414 derivative, expressing YPDI1	This study
p14EhPDI	pRS414 derivative, expressing *Eh*PDI (WT)	This study

### Plasmid shuffling and complementation assay

The ability of the amebic PDI to complement the yeast PDI1 mutation was assessed by a routine plasmid shuffling protocol 26. Competent RSA4‐16 cells were transfected with 1 μg of each PDI‐expressing plasmid (pYScPDI1, pYEhPDI, pYRM05, pYRM06, and pYRM15). Stable transformants harboring both URA3 and HIS3 plasmids were selected in synthetic dextrose (SD) medium (supplemented with G418) by their capability to proliferate in the absence of uracil and histidine. Independent Ura^+^ His^+^ colony‐forming units were cultured in minimal medium and then replica‐plated on selective medium, either lacking or containing 5‐FOA (0.1%), to induce the segregation of the URA3 plasmid. Positive complementants retained their viability in the presence of 5‐FOA. Genotypes were established by the analysis of auxotrophic markers, while elimination of pCT38 and retention of PDI‐expressing plasmids were confirmed by gene‐specific amplifications. Expression of *Eh*PDI was verified by immunoblot assays.

### Growth and DTT sensitivity phenotypes

Proficiency of the amebic PDI was tested by examining the growth and DTT sensitivity phenotypes exhibited by the positive complementants. Growth rates (and doubling times) were estimated from yeast cultures in yeast extract/peptone/dextrose (YPD) medium, measuring the OD_600_ every hour for an 8‐h period. As these showed an exponential increase, following a first‐order kinetics, the growth rate was obtained as the slope of the linear extent of plotting the ln(OD) with respect to time; however, doubling time was determined by dividing 0.693 by the slope. Sensitivity to the reductant agent DTT, a well‐known ER stress inducer that causes protein misfolding by disrupting disulfide bond formation 27, was assessed by the ability to grow in the presence of the reductant. Fresh cultures were used to normalize the cell number (OD_600_ = 1.0). From these, three serial dilutions were prepared (10^−1^ to 10^−3^) and 10 μL of each was spotted on selective medium, either lacking or containing 2 mm DTT.

### Temperature sensitivity phenotype

Competent W303‐1B Δpdi1/pCT37 cells were transfected with 2 μg of each PDI‐expressing plasmid (p14ScPDI1 and p14EhPDI). Stable transformants harboring both URA3 and TRP1 plasmids were selected by their capability to proliferate in the absence of uracil and tryptophan. Independent Ura^+^ Trp^+^ colony‐forming units were cultured in SGal/Raf medium and then replica‐plated on SC medium containing 5‐FOA (0.05%), to induce the segregation of the URA3 plasmid. Positive complementants retained their viability in the presence of 5‐FOA. Segregation of pCT37 and retention of PDI‐expressing plasmids were confirmed by PCR amplifications. The ability of the amebic PDI to support cell proliferation at 37 °C, a nonpermissive condition known as a promoter of thermal stress, which causes protein unfolding and triggers unfolded protein response (UPR) 22, was tested by examining the outcome after growing at that condition. Fresh cultures were used to normalize the cell number (OD_600_ = 1.0). From these, three serial dilutions were prepared (10^−1^ to 10^−3^) and 10 μL of each was spotted on selective medium.

### Data analysis

Unless otherwise mentioned, all data were from three independent experiments and represented as a mean ± standard error. Statistical analysis was performed using Prism® v.4 (GraphPad Software, San Diego, CA, USA).

## Results and Discussions

The premise that core functions are well conserved among eukaryotic cells 28 served as the basis to assume that yeast PDI1 mutant provides the cellular benefits needed to study the function of orthologous proteins. As PDI1 is essential (e.g., the null mutation leads to a lethal phenotype) 19, 20, any functional counterpart must be able to restore cell viability. Moreover, as *S. cerevisiae* remains as one of the major models of cell biology in eukaryotes 17, 29, the proficiency of any orthologous protein can be further examined at different cellular levels, as virtually all experimental systems are easier on yeast than on most other models 29, 30.

**Figure 2:**
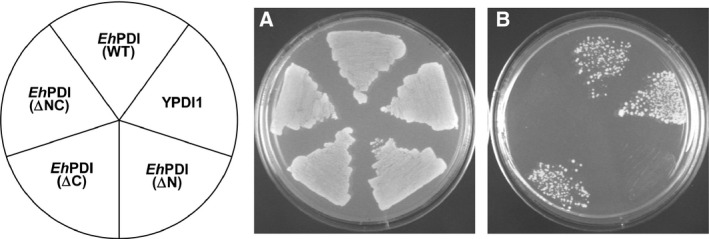
Functional complementation of yeast PDI1 mutation by the orthologous *Eh*PDI. RSA4‐16 (Δpdi1) cells expressing YPDI1 and *Eh*PDI (wild type or any other variant) were grown in selective medium, either lacking (A) or containing 0.1% 5‐FOA (B). The picture on the left represents the position of complementants on each plate. After 2–3 days of incubation, plates were photographed.

Because the precise intracellular location of PDI enzymes is important for proper function, *Eh*PDI was tagged with the yeast PDI1 signal peptide (N‐terminal) and the HDEL motif (C‐terminal) to ensure targeting into the ER compartment. The mutant strain RSA4‐16 [Δpdi1 (pCT38)] was used as a model to test the ability of the amebic PDI to complement the yeast PDI1 mutation and sustain cell viability. Through a plasmid shuffling assay, we found that *Eh*PDI (WT) supports growth on selective medium (Fig. 2), demonstrating that *Eh*PDI is actively expressed and complements the essential functions of PDI1. Conversely, no proliferation was observed in cells harboring the fully redox‐inactive enzyme *Eh*PDI (∆NC), indicating that it depends on functional thioredoxin‐like domains to assist the essential functions. We also tested the individual contribution of each thioredoxin‐like domain without the background of the other. Only *Eh*PDI (∆C) supported growth on selective medium (Fig. 2), unlike *Eh*PDI (∆N), suggesting that the N‐terminal thioredoxin‐like domain is the major contributor to the function.

**Figure 3:**
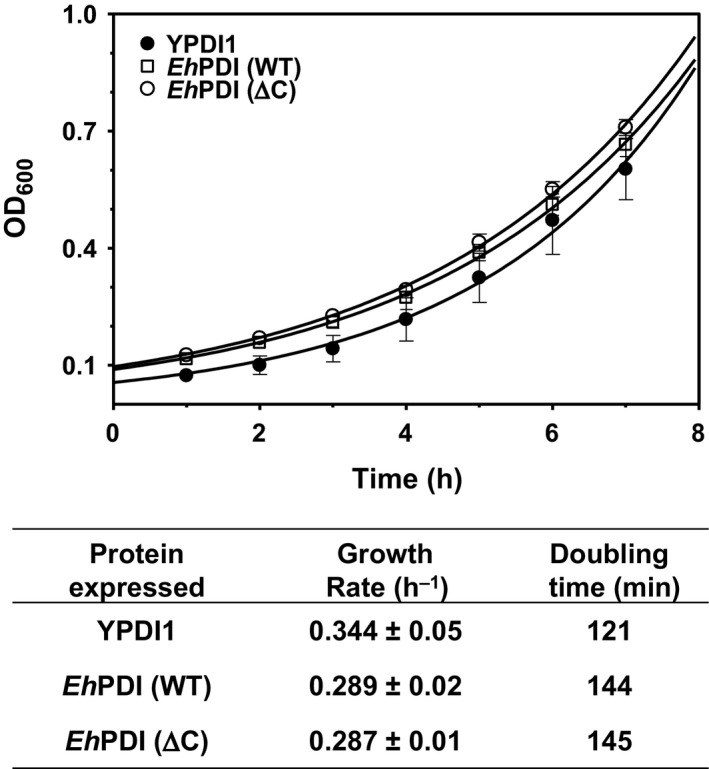
Growth kinetics of yeast PDI1 mutant expressing PDI proteins. Overnight cultures of positive complementants were diluted into fresh YPD medium (OD_600_ of 0.1) and then allowed to grow for 8 h. The OD_600_ was monitored every hour (upper panel). Each PDI is denoted by a distinct symbol. The growth rate and doubling time are also presented (bottom panel).

Proficiency of *Eh*PDI was further examined by determining the growth rate, which served as a quantitative estimation of the functional complementation. Prior to cell culture, the intracellular expression of *Eh*PDI was confirmed by immunoblotting (data not shown). Afterward, growth rates (and doubling times) were calculated from cultures expressing *Eh*PDI (WT) or *Eh*PDI (∆C). Interestingly, we found that *Eh*PDI supports growth in a similar way to that observed with yeast counterpart (Fig. 3). However, up to this point, complementation assays were carried out under permissive conditions for yeast proliferation, where the demand for functional chaperones and folding enzymes was physiologically regulated by protein homeostasis 31. Therefore, it is likely to assume that *Eh*PDI is functionally competent when subcellular conditions are stable.

In *S. cerevisiae*, DTT induces stressful conditions inside the ER compartment by reducing disulfide bonds in resident proteins 27. After establishment, ER stress triggers the UPR pathway, which is mediated by the kinase‐endoribonuclease IRE1 (sensor) and the transcription factor HAC1 (effector) 32. Consequently, the expression of several UPR‐target genes, such as BIP, PDI1, and FKB2, is induced by binding of HAC1 to specific regulatory elements, named UPRE, found in their promoters 33, 34, 35. The UPR is essential to protect cells against the lethal consequences of the ER stress: The accumulation of misfolded proteins leads to a prolonged UPR activation, which in turn causes oxidative stress and eventually cell death 36, 37, 38.

**Figure 4:**
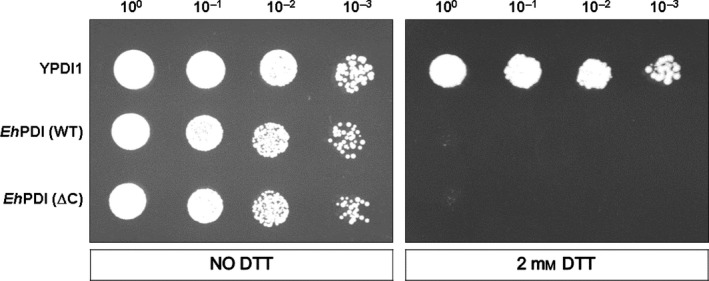
Sensitivity to ER stress induced by DTT. Overnight cultures of positive complementants (RSA4‐16, Δpdi1 cells) were diluted to an OD600 = 1.0, then serially diluted (down to 10–3) and spotted on selective medium, either lacking or containing 2 mm DTT. After 3–4 days of incubation, plates were photographed.

**Figure 5:**
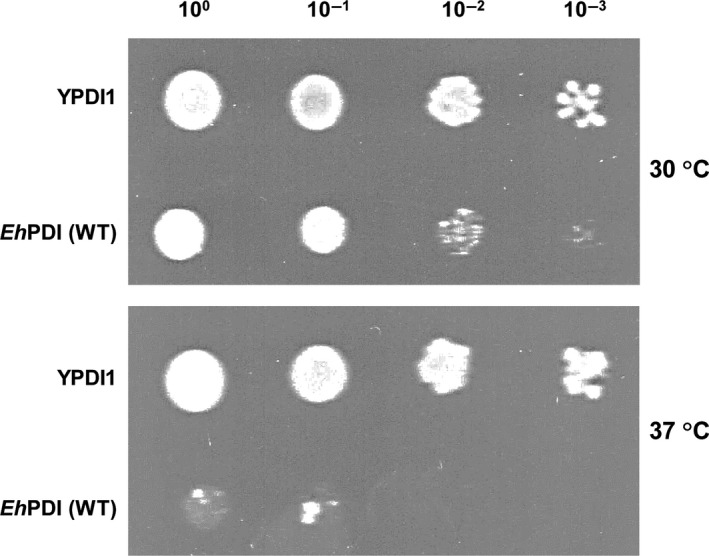
Temperature‐sensitivity. Overnight cultures of complementants (W303‐1B, Δpdi1 cells) expressing YPDI1 or *Eh*PDI (WT) were diluted to an OD600 = 1.0, then serially diluted (down to 10–3) and spotted on selective medium. After 3–4 days of incubation at 30 or 37 °C, plates were photographed.

The yeast PDI1 promoter contains two UPRE (CACCGTG and CACGTGTC) that have been identified as crucial for protein induction by ER stress 34, 39, which are similar to the canonical UPRE‐1 (CAGCGTG) and UPRE‐2 (CACGTGKC) elements, both *bona fide* binding sites of HAC1 40. As the expression of *Eh*PDI is controlled by the yeast PDI1 promoter, its functional proficiency can be further assessed by analyzing the resulted phenotype after the induction of ER stress. As expected, the yeast PDI1 supports the growth of RSA4‐16 cells in selective medium containing 2 mm DTT, confirming that it confers resistance to the prevailing conditions (Fig. 4). By contrast, no cell proliferation was observed when yeast harboring either *Eh*PDI (WT) or *Eh*PDI (∆C) was grown under similar conditions, suggesting that the amebic PDI is unable to sustain viability when protein homeostasis is altered. The latter observation was further validated under thermal stress conditions (Fig. 5), which disrupts cellular homeostasis by promoting the unfolding and denaturation of proteins 22, as a rather low propagation was noted in yeast expressing *Eh*PDI (WT).

## Conclusions

As closing remarks, our results suggest that *Eh*PDI is functionally active when physiological conditions are stable. However, when conditions are stressful; for instance, by the accumulation of misfolded proteins in the ER, its proficiency is exceeded, indicating incompetence to prevent unfolding, suppress aggregation, or assist refolding of proteins. Although this observation can be regarded as a preliminary data, our findings represent the initial step toward determining the participation of *Eh*PDI in cellular mechanisms related to protein homeostasis, a highly intricate mechanism. Notwithstanding, a complete functional analysis must be performed to assess the physiological role of this foldase within its cellular context. In this sense, a recent study reported the analysis of the *E. histolytica* gene expression under stressful conditions induced by nitric oxide (an immune component) 41. Interestingly, a dramatic upregulation of Hsp‐coding genes was observed, implying that nitric oxide triggers some stress response. Furthermore, a modulation of the expression of genes coding for enzymes involved in antioxidant pathways was detected (e.g., a peroxiredoxin and three iron‐sulfur flavoproteins were upregulated, while a thioredoxin and a disulfide isomerase were downregulated), suggesting that important changes in the redox balance might be occurring. Finally, it seems that *E. histolytica* has unique cellular responses to stressful conditions, which are of interest to understand how they participate during infection, and how to impair that ability and induce cell death.

## Author contributions

REM and MAR conceived and designed the study; analyzed and interpreted the data; and approved the final manuscript. REM performed the experiments and acquired the data. MAR wrote the first draft of the manuscript.
